# A case of polyploid utility in biocontrol: reproductively‐impaired triploid *Nasonia vitripennis* have high host‐killing ability

**DOI:** 10.1002/ps.8548

**Published:** 2024-12-23

**Authors:** Xuan Li, Kelley Leung

**Affiliations:** ^1^ Department of Entomology—College of Plant Protection Nanjing Agricultural University Nanjing China; ^2^ Groningen Institute for Evolutionary Life Sciences, University of Groningen Groningen The Netherlands; ^3^ Laboratory of Genetics Wageningen University and Research Wageningen The Netherlands

**Keywords:** triploid, diploid, sterile insect technique, parasitization, admixture, RNAi

## Abstract

**BACKGROUND:**

Intentionally impairing the fecundity of mass‐reared insects has important utility in controlling pest species. Typically, sterilized individuals are competed against wild counterparts, reducing pest population size. A novel consideration is creating biocontrol agents with lower reproductive capacity that are less likely to establish permanently or admix with wild populations, which are both emerging as legal barriers. Hymenopterans have diploid females, but archetypically infertile polyploid triploid females occur for various parasitoid species. As a first test of polyploid utility for these biocontrol concerns, we assessed the species with the best characterized polyploid biology, the gregarious idiobiont *Nasonia vitripennis*, for triploid female host‐killing ability on pupal blowfly hosts (*Calliphora vomitoria*).

**RESULTS:**

We examined four polyploid lines: the old Whiting polyploid line (WPL) derived from a spontaneous mutation, and new polyploid lines made through RNAi knockdown of sex determination genes *transformer*, *transformer‐2* and *wasp‐overruler‐of‐masculinization*. For diploid and triploid females of each polyploid line, and control diploids of the STDR and oyster lines used to maintain them, we measured lifetime number of hosts killed; lifetime number of hosts that produced at least one offspring; the percentage of the hosts killed and the percentage of hosts that produced offspring out of those offered; and lifespan. For all lines, triploids produced viable offspring in far fewer hosts than their diploid counterparts (≤70% less). Surprisingly though, they killed as many or more hosts than diploids over similar lifespans. The offspring production ability of the WPL triploid was half that of the other lines, but lines varied only slightly in the number of hosts killed (±10) among the polyploids.

**CONCLUSION:**

The ability of reproductively impaired triploids to kill as many hosts as fertile diploids demonstrate high biocontrol utility for polyploidized females, and downstream potential for reducing ecological risk. © 2024 The Author(s). *Pest Management Science* published by John Wiley & Sons Ltd on behalf of Society of Chemical Industry.

## Graphical abstract

Parasitization ability on blowfly pupae of polyploid (triploid) females of the model parasitoid *Nasonia vitripennis* was compared against diploid controls. Despite highly impaired reproduction, the infertile polyploids had parasitization ability equal to control diploids, demonstrating high utility of polyploid parasitoids for biocontrol, with less ecological risk of establishment or admixture.

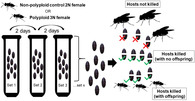

## 1 INTRODUCTION

Insects with intentionally impaired fecundity have a long history of use in pest management primarily through the sterile insect technique (SIT).^1^ Typically, males of problem species are sterilized through irradiation, then released to compete with wild counterparts for female mates. The wild females that mate with the SIT males produce no or fewer offspring, reducing the size of the pest population. Females have not been used in SIT for several reasons. Males tend to be the more promiscuous sex, so targeting their reproductive ability is more efficient for pest control. It also is more difficult to sterilize females completely, which translates to lower pest control, a higher amount of radiation needed for treatment and a higher health risk to human handlers.^1^, ^2^

An emergent possibility is adapting principles of SIT to optimize parasitoids used in biological control, the practice of using natural enemies to control pests. The parasitoid lifestyle is defined by obligate killing of a host used for offspring development.^3^ A major advantage of parasitoids is that they tend to have a specific host range and are therefore more specialized than predators. However, even though they are believed to have fewer nontarget effects, they still are subject to regulation based on their environmental risk.^4^, ^5^ Classical biological control strategies introduce new biocontrol agents with the aim of founding a permanent population which may have many adverse effects on local ecology. Even if the biocontrol agent species was previously already present in the area, a new strain can cause admixture.^6^, ^7^, ^8^, ^9^ Although sometimes beneficial for performance or establishment,^6^, ^8^, ^9^ this is problematic if the new strain dilutes natural genetic variation of local populations, or outcompetes native populations or subspecies. This is already on the radar of regulatory bodies and conservationists who advise that mass‐reared insect releases not be allowed if it cannot be shown that admixture would not occur.^9^, ^10^, ^11^, ^12^, ^13^ In an ideal scenario, released individuals could perform their applied function and then die out without reproducing. Parasitoid females with impaired fecundity but high host‐killing ability are thus desirable for biocontrol.

Interestingly, sterile or infertile polyploid females occur without irradiation in various parasitoid species.^14^, ^15^, ^16^ All parasitoid wasps, including those used in biocontrol, are hymenopterans, which all have haplodiploid sex determination.^14^ Unfertilized eggs develop into haploid males and fertilized eggs develop into diploid females. Polyploid diploid males and triploid females occur across numerous distantly related parasitoid wasp species.^14^, ^15^ So far, in all cases this can be attributed to perturbations of their sex determination mechanisms. In all but a few cases, triploid females are sterile or infertile.^17^, ^18^, ^19^ This greatly reduces their likelihood of establishing problematic permanent populations or interbreeding with local populations. However, it is unknown if triploid females perform just as well as diploid females in an applied setting, e.g. pest suppression.

Triploid parasitoid females thus must be tested for pest killing efficiency before they can be exploited for their sterility or infertility to reduce environmental risk. Therefore, we performed a case study using the gregarious idiobiont blowfly parasitoid *Nasonia vitripennis*. Species of *Nasonia* are employed in the biocontrol of stable flies,^20^, ^21^ and are often used as a general model for biological control study.^19^, ^22^ Triploid females of *N*. *vitripennis* have been known in laboratory studies for decades.^23^ Previous studies indicate that there are phenotypic differences (life history, cellular biology, expression) among polyploid lines, with effects of inbreeding *versus* outbreeding, and polyploidization method.^19^, ^24^, ^25^ We therefore conducted parasitization (host‐killing) assays across four different polyploid backgrounds, WPL and knockdown lines for the genes *transformer* (*tra*), *transformer‐2* (*tra2*) and *wasp‐overruler‐of‐masculinization* (*wom*), controlling for genetic variation though use of inbred lines. For triploid and diploid females of each line we measured: lifetime number of hosts parasitized (killed); lifetime number of hosts that produced at least one offspring; the percentage of the hosts killed or produced offspring out of those offered; and lifespan. We also measured these traits for diploid females of the mutant eye marker lines STDR and oyster used to maintain these polyploid lines, as additional diploid controls. Our aims were to identify whether triploids had comparable host‐killing ability to diploids, whether viable offspring production is related to host‐killing, and if polyploids of some lines had higher host‐killing ability than others. Knowledge of these life‐history traits are prerequisite for effective adaptation of sterile insect technique to use polyploid females for biocontrol.

## 2 MATERIALS AND METHODS

### 2.1 Generation and maintenance of polyploid lines

All lines were reared in an incubator under standard rearing conditions of 25 °C, 18 h:6 h, light:dark and ~55% relative humidity (RH), on commercial *Calliphora vomitoria* blowfly pupal hosts (Titus Blom, Groningen, the Netherlands). The Whiting polyploid line (WPL) was acquired from the John H. Werren laboratory (Rochester, New York, USA). The WPL breeding scheme is fully described elsewhere,^19^, ^24^, ^26^ but in brief, WPL carries two complementary eye mutation markers, *oyster* (*oy*) and STDR (*st*). Virgin triploid females with two copies of the STDR allele and one *oy* allele (*st* +/ *st* +/ + *oy*) have a wild‐type (WT) purple‐eyed phenotype. They were hosted to produce red‐eyed haploid (*st* +) and diploid (*st* +/*st* +) males, which were discarded; and purple‐eyed diploid (*st* +/ + *oy*) males that were crossed to purebred red‐eyed diploid females of the purebred STDR line (*st* +/*st* +) to produce triploid females for assays of this study, per the usual WPL maintenance protocol. Nonpolyploid diploid female controls (*st* +/ *st* +) for this line were produced through the purebred STDR line (an inbred mutant line that also is maintained separate from WPL).

**Figure 1:**
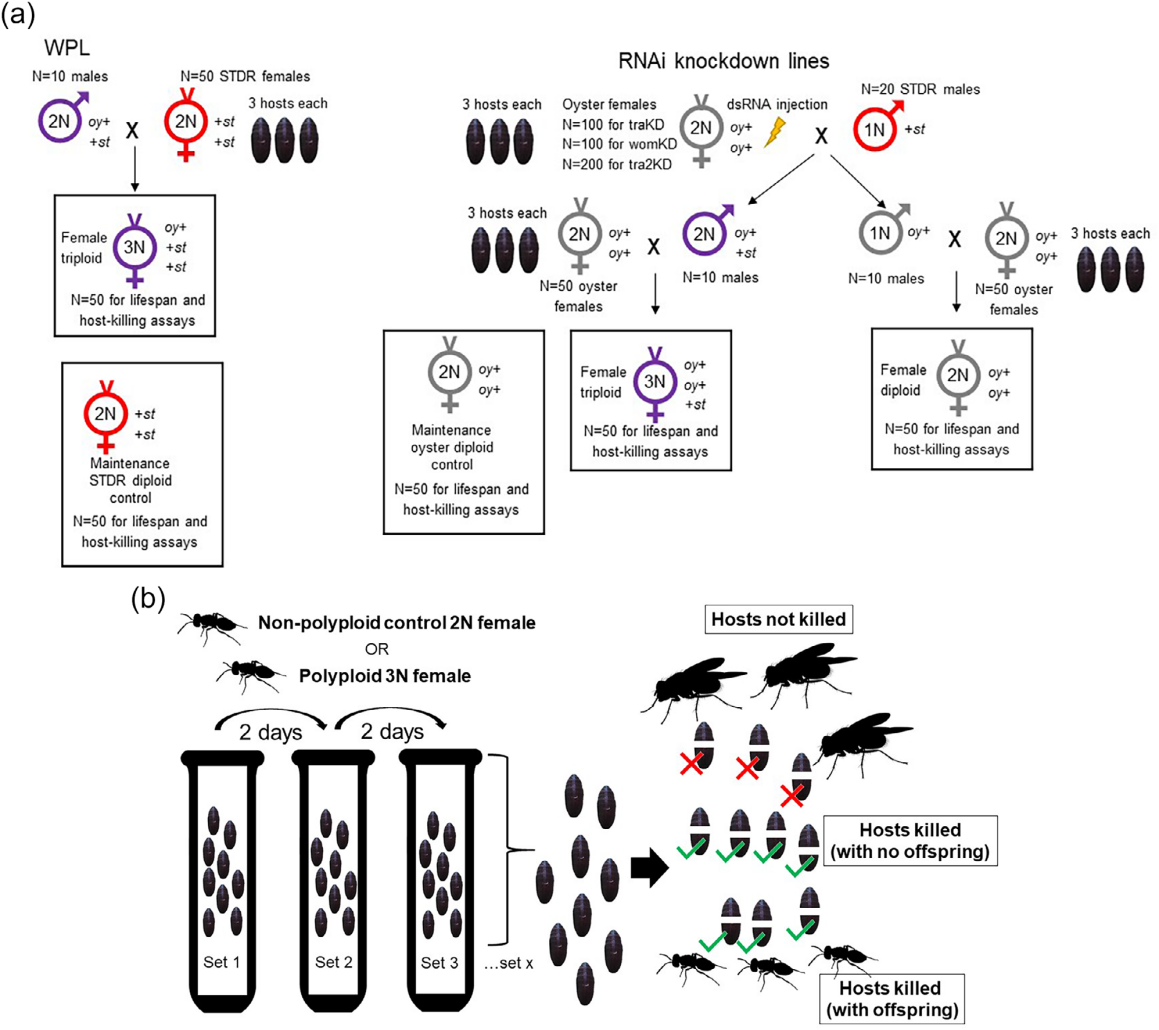
Production of experimental females and lifespan and host‐killing assays (a) Diploid (polyploid) males needed for crosses to produce experimental triploid females in the parasitization assays were produced from virgin triploid mothers that are isogenic within their own line. For the WPL, 10 diploid males were mass‐mated to 50 maintenance STDR red‐eyed females that were then given three hosts each. From their pooled female offspring, 50 random dark‐eyed triploids were used for the experiment. 50 females from STDR were used as an additional maintenance line diploid control. Owing to variation in knockdown success across lines, different numbers of virgin oyster mothers were injected hosted to obtain diploid males; *N* = 100 for the traKD and womKD lines; and *N* = 200 for the tra2KD line. These mothers were mass‐mated with *N* = 20 STDR males and given three hosts each. From this mass mating, 10 randomly collected dark‐eyed diploid sons were mass‐mated to 50 maintenance oyster females that were then given three hosts each. From their pooled female offspring 50 random dark‐eyed triploids were used for the experiment. From this same mass mating, 10 randomly collected grey‐eyed haploid males were mass‐mated to 50 maintenance oyster females that were then given three hosts each. From their pooled female offspring 50 random grey‐eyed diploid controls were used for the experiment. 50 females from oyster were used as an additional maintenance line diploid control. (Figures adapted from Sivaprakasham Murugesan *et al*.^25^) For (b) host‐killing and lifespan assays, females were transferred to a fresh set of hosts every 2 days until death, with date of death being measured for lifespan. Each set was then scored for number of hosts that were not killed (developed into an adult fly), hosts that were killed but did not produce offspring, and hosts that were killed and produced at least one offspring (see methods for more details).

RNAi knockdown lines were produced for *transformer* (*tra*),^27^ *transformer‐2* (*tra2*)^28^ and *wasp overruler‐of‐masculinization* (*wom*)^29^ (see references for primer sequences, dsRNA synthesis protocols, and working concentrations for RNAi knockdown). A full account of knockdown line maintenance has been given previously.^25^ Briefly here, purebred grey‐eyed *oyster* line females, (an inbred mutant line that also is maintained separate from the knocked‐down lines), (+ *oy/* + *oy*) were microinjected with dsRNA of the target gene at the white pupal stage. These females were allowed to develop and eclose as adults, then mated with red‐eyed STDR males (*st* +). The knocked‐down lines all neutralize a feminization factor, so diploidized purple‐eyed males (+ *oy*/*st* +) were produced, in addition to grey‐eyed haploid (+ *oy*) males from unfertilized eggs. The diploid males were crossed to purebred grey‐eyed virgin diploid females of the *oy* line to produce triploid purpled‐eyed females (*st* +/ + *oy*/ + *oy*) for assays. The grey‐eyed haploid males also were crossed to *oy* females, to produce nonpolyploid control diploids (+ *oy*/ + *oy*). These RNAi knockdown lines can be maintained indefinitely,^25^ but here, only F_2_ females (the earliest female generation) were assessed. A further nonpolyploid control for the triploids of the knockdown lines were diploid females from the *oy* line itself. For a fuller description and schematic diagram on the generation of test females for parasitization assays, see Fig. 1(a).

### 2.2 Host‐killing and lifespan assays

All test females were collected from hostings of a single mother on three hosts. As 1‐day‐old virgins, test females were individually given 10 fresh hosts in a small plastic tube plugged with cotton. Every 2 days they were transferred to another set of 10 fresh hosts until death (i.e. an intended excess of hosts that could possibly be used for feeding or reproduction). Lifespan was measured up to the day a dead female was observed during host transfer. Host sets were kept at standard incubator conditions for ≥2 months, to far exceed both the 2‐week life cycle of *N*. *vitripennis* and the 3‐week life cycle of *C*. *vomitoria* blowfly hosts. Host sets were then scored for the number of hosts that were killed (did not develop further and eclose into an adult fly) and the number of hosts that produced offspring (one or more *N*. *vitripennis* offspring that developed to at least the larval stage). All hosts that did not develop into flies were coded as ‘host‐killed’, but not all of these had offspring; therefore, the number of hosts coded as ‘hosts with offspring’ reflects a subset of the number coded as ‘hosts‐killed’. Offspring hosts were recognized either by the presence of holes on the cuticle, indicating that mature *Nasonia* chewed their way out from within, or by cracking open the host and finding dead adult or larval offspring. As escape by chewing is more effective with a larger number of adult siblings, the cracking method of hosts is essential to score offspring production for polyploids in particular. Their low reproductive ability means they often do not produce enough viable offspring to chew escape holes. As females were exposed to different total number of hosts because some lived longer than others, we also looked at the percentage of hosts killed and produced offspring out of those offered. For a schematic representation of these assays, see Fig. 1(b).

### 2.3 Plotting and statistics

All plotting and statistics were performed with R v4.2.1. Box plots were generated with R package ggplot2 v3.4.4.^29^ For all lines, triploids were compared against their diploid controls, to establish how polyploidy impacts traits of lifespan, total number of hosts killed, total number of hosts that produced at least one offspring, and percentages of hosts offered that were killed or produced offspring. We used generalized mixed models (GLMM) with binomial errors (glmer) to test whether triploid females and diploid females differed in abilities of host‐killing and offspring‐producing respectively, using the packages lme4. GLMM was used to test whether triploid females and diploid females differed in lifespan using the packages glmmtmb,^30^ with a negative binomial distribution (nbinom2) selected to appropriately model the count nature of the lifespan data and account for any overdispersion in the response variable. Then, triploids of all lines were compared to each other, to test differences in polyploid performance in different lines. For two‐group comparisons [e.g. the STDR (diploid line) *versus* WPL (triploid line)] we used Wilcoxon rank sum and Signed rank tests. For three or more group comparisons, Kruskal–Wallis rank sum tests were first applied to test for significant differences among groups. Following a significant Kruskal–Wallis rank sum result, we performed *post hoc* Dunn's Kruskal–Wallis multiple comparisons (from the R package pmcmrplus v1.9.10) to identify groups with significant pairwise differences.

## 3 RESULTS

### 3.1 Triploid lifespan is comparable to diploid lifespan

**Figure 2:**
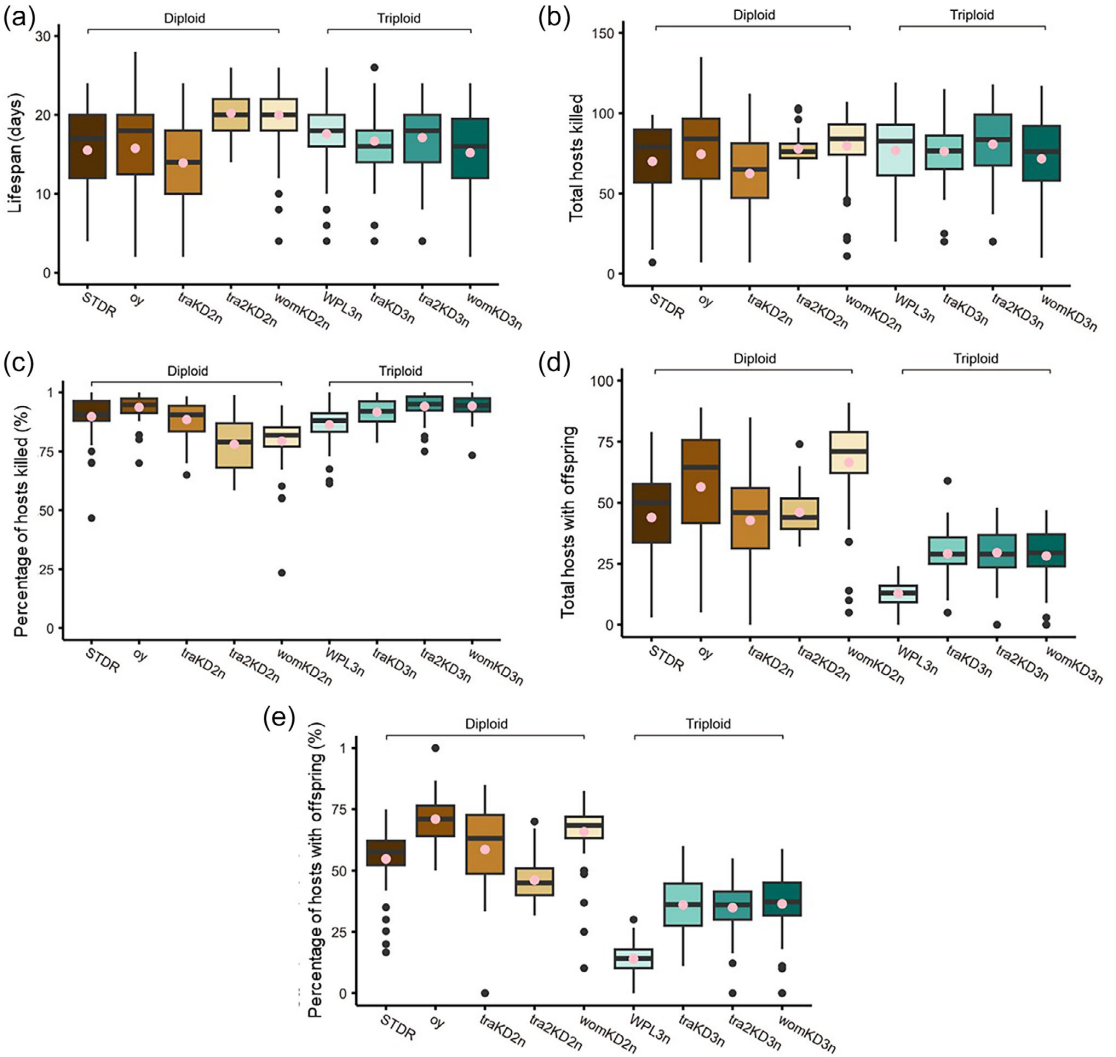
Lifespan, hosts killed, and hosts with offspring of diploid and triploid females of polyploid lines. (a) Lifespan indicates the number of days the female survived following first hosting. (b) Total number of hosts‐killed by the female. (c) Percentage of hosts‐killed out of those offered (10 every 2 days). (d) Total number of hosts that resulted in offspring production. (e) Percentage of hosts that resulted in offspring production. Brown‐toned data indicate the diploid (nonpolyploid) groups. Green‐toned data indicate the triploid (polyploid) groups. Pink dots represent means and lines in boxes represent medians.

Overall there was no significant difference in lifespan between triploids and diploids (Table 1). However, we observed some variations among tested lines. The lifespans of WPL triploids and their STDR diploid controls used for line maintenance were not significantly different [Fig. 2(a); Tables 2 and 3]. Diploids of the line used to maintain all the other polyploid lines (oy) also did not have significantly different lifespans from their triploid counterparts of the traKD, tra2KD and womKD lines. Within the polyploid lines, however, diploids and triploids had some differences. For the traKD line for example, the lifespan of diploids (traKD2n), was significantly shorter than that of triploids (traKD3n) [Fig. 2(a); Tables 2, 4 and 5]. In regards to diploid *versus* triploid lifespan for the tra2KD lines and womKD lines, diploids (traKD2n and womKD2n) had significantly longer lifespans than triploids (traKD3n and womKD3n). Furthermore, the lifespans of traKD2n and womKD2n diploid were significantly longer than all other tested diploids [STDR, oy and *tra*KD 2n, Tables 2, 4 and 5]. Triploid lifespans for all polyploid lines were not significantly different [Fig. 2(a); Tables 4 and 5]. Overall, there was more variation in lifespan among diploids of different lines than there was for triploids of different lines. In three of five lines, the triploid female lifespan was not significantly shorter than the diploid counterpart.

**Table 1:** Mixed model results for the effect of ploidy on three traits of females: lifespan, hosts‐killed and hosts with offspring

Response	Intercept	Coefficient	*z*‐value	*P*‐value
Lifespan	2.83 ± 0.05	−0.02 ± 0.08	−0.22	0.83
Hosts killed	1.97 ± 0.23	0.48 ± 0.34	1.41	0.16
Hosts with offspring	0.44 ± 0.20	−1.33 ± 0.30	−4.49	7.01e‐06***

*Note*: Line difference was included as a random effect. Bold text signifies significance (***, *P* < 0.05).

**Table 2:** Lifespan, hosts killed, and hosts with offspring for diploid (2n) and triploids (3n) of different lines

Line (*N* = 50 ea)	Mean ± SD				
	Lifespan (days)	Total hosts‐killed	Total hosts with offspring	Percentage of hosts‐killed	Percentage of hosts with offspring
STDR	15.33 ± 5.77	69.02 ± 26.23	43.25 ± 19.18	89.73 ± 9.48%	54.76 ± 12.64%
oy	15.76 ± 6.27	74.48 ± 30.19	56.50 ± 23.70	93.69 ± 5.46%	71.00 ± 9.34%
traKD2n	13.92 ± 4.95	62.40 ± 23.34	42.76 ± 19.29	88.47 ± 7.76%	58.59 ± 17.95%
tra2KD2n	20.20 ± 2.95	77.80 ± 9.38	46.08 ± 8.83	78.00 ± 10.54%	46.09 ± 8.72%
womKD2n	19.96 ± 4.45	79.62 ± 21.25	66.52 ± 19.33	79.33 ± 11.87%	65.77 ± 13.18%
WPL3n	17.60 ± 5.03	76.82 ± 24.13	12.84 ± 6.11	87.30 ± 9.30%	14.59 ± 6.13%
traKD3n	16.68 ± 4.51	76.28 ± 20.09	29.12 ± 9.97	92.02 ± 6.25%	36.00 ± 11.70%
tra2KD3n	17.12 ± 5.05	80.56 ± 24.65	29.56 ± 10.39	94.10 ± 5.47%	34.87 ± 10.05%

**Table 3:** Wilcoxon rank‐sum test between STDR diploids and WPL3n triploid for lifespan, hosts killed and hosts with offspring

Category	*W*‐value	*P*‐value
Lifespan	1477.5	3.38E‐01
Total hosts‐killed	1389.5	1.14E‐01
Total hosts with offspring	255	**6.87E‐03**
Percentage of hosts‐killed	857.5	**6.92E‐12**
Percentage of hosts with offspring	26	**2.20E‐16**

*Note*: Bold text signifies significance (*P* < 0.05).

**Table 4:** Kruskal–Wallis *H*‐test for lifespan, hosts killed and hosts with offspring

Compared group	oy, traKD2n, traKD3n			oy, tra2KD2n, tra2KD3n			oy, traKD2n, traKD3n			WPL3n, traKD3n, tra2KD3n, womKD3n		
Category	*H*‐value	df	*P*‐value	*H*‐value	df	*P*‐value	*H*‐value	df	*P*‐value	*H*‐value	df	*P*‐value
Lifespan (days)	8.3185	2	**1.56E‐02**	17.07	2	**1.96E‐04**	27.69	2	**9.71E‐07**	7.5723	3	5.57E‐02
Total hosts‐killed	10.72	2	**4.70E‐03**	1.7105	2	4.25E‐01	3.9684	2	1.38E‐01	3.9816	3	2.64E‐01
Total hosts with offspring	39.956	2	**2.11E‐09**	54.613	2	**1.38E‐12**	62.774	2	**2.34E‐14**	74.581	3	**4.46E‐16**
Percentage of hosts‐killed	15.849	2	**3.62E‐04**	67.918	2	**1.79E‐15**	75.049	2	**2.20E‐16**	41.64	3	**4.78E‐09**
Percentage of hosts with offspring	84.71	2	**2.20E‐16**	106.43	2	**2.20E‐16**	89.588	2	**2.20E‐16**	89.041	3	**2.20E‐16**

*Note*: Bold text signifies significance (*P* < 0.05).

**Table 5:** Dunn's multiple comparison *post hoc* tests for significant Kruskal–Wallis *H*‐tests

Category	Lifespan (days)		Total hosts killed		Total hosts with offspring		Percentage of hosts killed		Percentage of hosts with offspring	
Comparison	*Z*	*P*_adj_	*Z*	*P*_adj_	*Z*	*P*_adj_	*Z*	*P*_adj_	*Z*	*P*_adj_
oy‐traKD2n	2.31	**3.64E‐02**	3.25	**7.99E‐03**	3.46	**8.66E‐04**	2.77	**7.95E‐03**	2.94	**4.66E‐03**
oy‐traKD3n	0.01	9.94E‐01	0.65	6.03E‐01	1.57	1.43E‐01	6.66	**1.15E‐10**	9.55	**1.39E‐20**
traKD2n‐traKD3n	−2.30	**3.42E‐02**	−2.60	**3.86E‐02**	−1.89	8.25E‐02	3.89	**1.72E‐04**	6.61	**9.93E‐11**
oy‐tra2KD2n	−3.95	**2.05E‐04**	0.50	6.78E‐01	7.66	**8.04E‐14**	1.34	2.09E‐01	6.50	**1.85E‐10**
oy‐tra2KD3n	−0.85	4.34E‐01	−0.67	6.56E‐01	−0.37	8.29E‐01	6.39	**5.67E‐10**	9.90	**9.04E‐22**
tra2KD2n‐tra2KD3n	3.10	**3.75E‐03**	−1.18	3.85E‐01	−8.03	**2.09E‐14**	5.05	**1.02E‐06**	3.40	**1.11E‐03**
oy‐womKD2n	−3.98	**2.40E‐04**	−0.96	5.08E‐01	7.43	**3.66E‐13**	−2.09	**4.77E‐02**	1.23	2.56E‐01
oy‐womKD3n	1.10	3.56E‐01	1.32	3.30E‐01	−0.27	8.72E‐01	6.74	**8.08E‐11**	9.16	**3.52E‐19**
womKD2n‐womKD3n	5.08	**2.62E‐06**	2.27	8.07E‐02	−7.70	**6.99E‐14**	8.84	**2.07E‐17**	7.93	**7.41E‐15**
tra2KD3n‐traKD3n	0.86	4.70E‐01	1.21	4.54E‐01	2.21	**4.06E‐02**	0.28	9.37E‐01	−0.23	8.20E‐01
tra2KD3n‐womKD3n	2.01	1.34E‐01	1.92	3.27E‐01	0.06	9.55E‐01	0.42	1.00E+00	−0.68	7.42E‐01
traKD3n‐womKD3n	1.15	3.76E‐01	0.72	7.12E‐01	−2.15	**3.75E‐02**	0.14	8.88E‐01	−0.46	7.78E‐01
tra2KD3n‐WPL3n	−0.58	5.64E‐01	0.68	5.98E‐01	5.61	**1.24E‐07**	7.28	**2.08E‐12**	7.38	**3.16E‐13**
traKD3n‐WPL3n	−1.43	3.03E‐01	−0.53	5.96E‐01	3.40	**1.37E‐03**	7.00	**7.85E‐12**	7.61	**8.40E‐14**
womKD3n‐WPL3n	−2.58	5.87E‐02	−1.25	6.38E‐01	5.55	**8.61E‐08**	6.86	**1.42E‐11**	8.06	**4.45E‐15**

*Note*: Bold text signifies significance, adjusted *P*‐value (*P*_adj_ < 0.05).

### 3.2 Numbers of hosts killed are higher in triploids than diploids, and are similar among lines

When we tested the host‐killing ability of diploids *versus* triploids, total number of hosts killed did not differ significantly [Fig. 2(b); Table 1], nor did the percentage of hosts killed out of those offered, with a few exceptions [Tables 2, 3, 4, 5]. Only diploids of traKD2n killed significantly fewer hosts than the triploid counterparts of their line, traKD3n. As for the percentage of hosts killed, females from all lines had a high, similar rate. The average percentage of hosts killed across female groups ranged from ~78% (tra2KD2n) to 94.21% (womKD3n) [Fig. 2(c); Table 2]. When comparing triploid females to diploid females, only triploids from WPL had a percentage of hosts‐killed that was significantly lower than the diploid females from STDR line, although the average rates for these two lines were similar (87.3 and 89.7%, respectively; Table 1). In comparisons of other polyploid lines, triploid females either had no differences or significantly higher percentages of hosts killed compared to their diploid female counterparts [Fig. 2(c); Tables 2, 3, 4, 5]. Together with comparisons of hosts‐killed number, those comparisons show that the killing ability of triploid females is comparable to that of diploid females or slightly better.

### 3.3 Reproductive rates of triploids are significantly lower than those of diploids

All triploids, regardless of specific line, had far fewer hosts producing offspring compared to diploids of their lines [Fig. 2(d); Table 2]. As expected, the same trend existed for percentages of hosts that produced offspring, with percentages being much higher for all diploids than for all triploids [Fig. 2(e); Table 2]. This indicates a general reduced reproduction ability in triploid females. Interestingly, there was variation in triploid reproduction ability among lines. Triploid individuals from the WPL had significantly fewer total hosts that produced offspring, and a lower percentage of hosts that produced offspring, than the triploids of the other three lines [Fig. 2(d),(e); Tables 2, 4, 5]. Triploid females from traKD3n, tra2KD3n and womKD3n did not significantly differ from each other for these two data types. This suggests triploid females of different polyploid lines vary in reproductive ability, with WPL being particularly divergent.

## 4 DISCUSSION

In the current study, we investigated the potential of triploid parasitoid wasps as biocontrol agents. Using *N*. *vitripennis* as a model, we aimed to show whether triploid individuals have the same host‐killing ability as diploid individuals while reproducing significantly less. Our results showed that triploid females had the same killing ability as their diploid controls, or slightly better. Despite the polyploids having on average a shorter lifespan than diploids, the lifetime number of hosts killed was similar across nonpolyploid diploids and polyploid triploids of lines (60–80 hosts). By contrast, triploids had far fewer hosts killed that yielded at least one offspring to the larval stage or further. These results indicated that triploid parasitoid females can provide sufficient killing efficiency while being likely to cause lower environmental risk upon release (i.e. less disturbance to wild populations of the same species).

The expected rate of euploid egg production by triploid *Nasonia* is 1/32, given that *Nasonia* has five chromosomes (most are aneuploid and shrivel). In previous studies, triploid females of WPL matched this rate of offspring production exactly,^19^, ^23^ but those of the traKD, tra2KD and womKD have a much higher fecundity (this study, unpublished data). The unusually high rate of viable offspring production in these RNAi knockdown lines may be to the result of partially viable aneuploids (unpublished data). However, it is unclear whether the females of the different polyploid lines vary in how many eggs clutches they oviposit. Although this can be scored by cracking open hosts and counting freshly deposited eggs (i.e. before they shrivel from aneuploidy), in this study it would have prevented accurate scoring of host‐killing. In the future it would be interesting to measure this trait, as it would indicate whether the higher fecundity of the RNAi knockdown lines is a consequence of more eggs being laid overall, a lower rate of inviable aneuploidy (same number of eggs laid, but more develop to adulthood), or both.

An interesting finding is that the reproductive impairment was particularly pronounced for the WPL, which had offspring in half as many of its hosts killed as the RNAi knockdown lines. Notably, WPL is a long‐term inbred strain which is expected to have lower genomic heterozygosity in the population. This variation between WPL, an old and long‐maintained line, versus the early F_2_ generation of newly created lines is possibly explained by the genetic background differences among the lines. Another hint that genetic background influences triploid life history and host‐killing traits comes from a previous study, in which WPL triploids had significantly lower numbers of hosts killed and shorter lifespan than diploid females, but the diploid females were of a different background than those used here (an internal inbred cross, and a genetically variable outcross).^19^ In this current study, when we compared overall performance of WPL triploids to STDR diploids, the differences were not as pronounced. Such findings demonstrated that it is necessary to test targeted traits in various genetic backgrounds to optimize triploid development for biocontrol function.

The reduced offspring production was expected of the polyploids, which are archetypically infertile, yet the high host‐killing ability of the polyploids was unexpected. Like other parasitoids, the *Nasonia* parasitization process has multiple steps, including envenomation, developmental arrest of the host, host‐feeding by the mother and host‐consumption by offspring.^31^, ^32^, ^33^ Although the individual contribution of each step to host‐killing is difficult to assess,^34^ it is certain that triploid‐stung hosts have reduced larval feeding. This would seemingly lessen the efficacy of at least one parasitization step. That the triploids had such high host‐killing ability may be to the result of high performance for other parts of the parasitization process.

Relatedly, one logistic challenge in using polyploid lines for biocontrol is whether females always need to be laboriously generated *de novo* with RNAi treatment, or if they can be bred continuously in stable lines. For the *Nasonia* system, a small number of chromosomes allows for enough incidental euploids for polyploidy to be stably inherited, as is evident in the continual production of WPL for decades.^23^ The same applies for the RNAi lines. Although we only assessed the first female generation for this study, they have been maintained continuously, and as of fall 2024, they are in the F_30_ generation. Although these lines were all inbred to eliminate background effects of genetic variation, this also suggests the potential of outbred stable lines. This would enable the highly desirable option of selecting triploid females. Whether long‐lasting polyploid lines are possible in other parasitoid species depends on multiple factors including whether they have a low enough chromosome number (which has been measured to range from *N* = 3 to *N* = 24 for parasitoids^35^), sufficiently high egg production rate and any aneuploid survival (as might be occurring for the RNAi knockdown lines of this study).

Our study supports further exploration of polyploid parasitoid usage in biocontrol, yet some other important caveats need to be considered. For instance, taxon choice is important. *Nasonia* spp. are gregarious (more than one offspring developing from a single host) and idiobionts (kill the host immediately upon parasitization).^31^, ^32^ The success of polyploids may only be tentatively expected of biocontrol agents of the same class. For species that are koinobiont, which allows the host to develop further before it is later killed by offspring feeding and development,^36^ a threshold level of offspring feeding may be necessary to kill the host. Thus, triploid koinobionts with reduced offspring production would be likely be less useful for biocontrol. Another difference may be whether the parasitoid is a gregarious or solitary species. The single eggs of solitary species are sometimes encapsulated by the immune system of the host, allowing the host to evade death.^37^ To have even lower oviposition ability of viable eggs may reduce solitary parasitoid kill rate. Thus, triploids of gregarious idiobionts in the same superfamily as *Nasonia*, such as *Trichogramma* spp. (important biocontrol agents of numerous lepidopteran pests globally^38^), have higher potential to be developed as biocontrol agents with less ecological risk.

Using *N*. *vitripennis*, we demonstrated that triploid individuals can be obtained either from spontaneous mutation or generated via RNAi targeting of key genes in sex‐determining pathways. More methods can be applied to induce triploid females. For instance, triploid queens of *Bombus terrestris* have been produced with CO_2_ narcosis^39^ and it is potentially possible to produce a large volume of triploid workers. We also have confirmed that polyploidy can be induced with colchicine inhibition of meiotic segregation in insects, using this *Nasonia* system.^25^ In commercial bee species such as *Apis* spp. and *Bombus* spp., inbreeding can lead to production of diploid males as well (through their shared complementary sex determination system).^15^, ^16^ In species for which endosymbiotic bacteria interfere with their genetic feminization pathways (e.g. *Leptopolina* spp. and *Asobara* spp.), diploid males can be produced via antibiotic curing.^40^, ^41^, ^42^ The next step of producing triploid females requires diploid male fecundity, which is not typical. But notably, none of the aforementioned techniques involve genetically modified organisms (GMO). This includes the RNA interference method used in this study, which is not legally classified as genetic modification as it prevents mRNA expression rather than modifying the nuclear genome. This makes it compatible with anti‐GMO approaches often built into biocontrol and integrated pest management programs.^22^, ^43^, ^44^

## 5 CONCLUSION

As polyploid incidence, behavior and fitness has been studied for only a small portion of the vast diversity of Hymenoptera, it is likely to be a matter of time before more cases of more triploids are documented (e.g. the highly functional triploid females of *Tapinoma erraticum* ^45^ ants, *Polistes* ^17^ vespids and the parasitoid *Cotesia vestalis* ^46^). Our results of a consistently strong parasitization ability of polyploid females here is a first indication for their utility in biological control. Now that we have tested the concept of polyploids having high host‐killing in the laboratory despite reduced reproduction, fuller study is needed to affirm its utility in more complex and applied field settings. Now that we have tested the concept of polyploids having high host‐killing in the laboratory setting despite reduced reproduction, more comprehensive study is needed to affirm its utility in more complex and applied field settings. For example, although we provided an excess of hosts in this study, for this species (*N*. *vitripennis*) and other parasitoids, an excessively high female density for a limited number of hosts results in multiple females ovipositing in the same host.^47^ This might increase ecological risk because there would be more individuals to assist with chewing exit holes in the host. This would allow more viable offspring to enter the field than if there had been oviposition by a single female whose few viable offspring might have died inside the host. Optimization of parasitoid‐to‐host ratio would thus need to be accounted for before field release. In future, such cumulative knowledge will be likely to furnish a rich world of commercial hymenopteran polyploid utility to explore.

## CONFLICT OF INTEREST

The authors have no conflict of interest to declare.

## Data Availability

The data that support the findings of this study are available from the corresponding author upon reasonable request.
